# Severe Neurotoxicity due to *Atropa belladonna* Poisoning: A Case Report and Literature Review

**DOI:** 10.1155/2024/5411258

**Published:** 2024-09-26

**Authors:** Seyed Javad Boskabadi, Sima Ramezaninejad, Zakaria Zakariaei

**Affiliations:** ^1^ Student Research Committee Pharmaceutical Sciences Research Center Faculty of Pharmacy Mazandaran University of Medical Sciences, Sari, Iran; ^2^ Toxicology and Forensic Medicine Division Mazandaran Registry Center for Opioids Poisoning Antimicrobial Resistance Research Center Imam Khomeini Hospital Mazandaran University of Medical Sciences, Sari, Iran

## Abstract

*Atropa belladonna* (*A. belladonna*), commonly known as deadly nightshade, is a poisonous plant belonging to the Solanaceae family. The toxic effects of *A. belladonna* are attributable to its alkaloid content, which possesses potent anticholinergic properties. These alkaloids are responsible for the plant's toxicity and can cause a range of adverse effects in humans and animals upon ingestion or contact. In this report, we describe two atypical cases of *A. belladonna* poisoning resulting from accidental ingestion of the plant's raw leaves, which were referred to the emergency room of a poisoning center in northern Iran. Both patients presented with symptoms of anticholinergic toxicity, including dry mouth, mydriasis, tachycardia, and delirium. The patients were managed conservatively with supportive measures, including hydration and administration of benzodiazepines to control agitation and delirium. With appropriate treatment, both patients showed improvement and were discharged from the hospital. *A. belladonna* intoxication is associated with a range of clinical manifestations, primarily due to its neurotoxic effects. These manifestations may include flushing, mydriasis, tachycardia, ataxia, agitation, delirium, and urinary retention. The severity of symptoms can vary depending on the amount of the toxin ingested and the individual's susceptibility. In severe cases, *A. belladonna* toxicity can lead to seizures, coma, and even death. These cases highlight the importance of awareness regarding the potential toxicity of *A. belladonna* and the necessity of prompt and appropriate management of its toxicity. In severe cases, physostigmine may be considered for the treatment of neurological symptoms due to the plant's anticholinergic effects.

## 1. Introduction

*Atropa belladonna (A. belladonna),* commonly known as deadly nightshade, is a poisonous plant belonging to the Solanaceae family. The plant derives its name from the Italian words “*bella*” and “*donna*,” which mean “beautiful lady.” This nomenclature may have arisen from the use of the plant to dilate the pupils of women, which was once considered a desirable cosmetic effect [1]. *A. belladonna* roots, leaves, and fruits contain different alkaloids, including atropine, hyoscyamine, and scopolamine. These alkaloids are responsible for the anticholinergic toxicity of the plant [2]. After the plant is ingested, most of the scopolamine is metabolized to atropine. Therefore, atropine and scopolamine are the main alkaloids in *A. belladonna* [3]. Previous research has shown that the leaves of *A. belladonna* can be used in the treatment of gastrointestinal spasmodic disorders. In particular, the medication known as belladonna-phenobarbital (*belladonna* PB), containing a blend of atropine, hyoscyamine, and hyoscine alkaloids, has been formulated for this specific therapeutic purpose. In addition, it is used in traditional medicine for the treatment of asthma and bronchitis [4].

Anticholinergic toxicity can present with a range of clinical manifestations, including flushing, mydriasis, tachycardia, ataxia, agitation, delirium, and urinary retention. *A. belladonna*, which has potent neurotoxic and deliriant effects, may cause a similar constellation of symptoms, such as lethargy, excitation, blurred vision, incoherent speech, pupil dilation, and coma [5]. The main cause of neurotoxicity is due to the presence of antimuscarinic alkaloids in this plant. Atropine and scopolamine are known as antimuscarinic agents. They block the muscarinic receptor, resulting in neurotoxic effects such as memory dysfunction, disorientation, sedation, and delirium [6].

*A. belladonna* toxicity has been documented in various age groups, although it is primarily used by adults seeking its deliriant effects, and accidental ingestion by adults is uncommon [7]. Despite numerous reports on the toxicity and therapeutic applications of *A. belladonna*, there remains a paucity of data regarding its neurological toxicity. In this report, we present two atypical cases of *A. belladonna* intoxication resulting from accidental ingestion of the plant's raw leaves, which were referred to the emergency room (ER) of a poisoning center in northern Iran.

## 2. Case Presentation

### 2.1. Case 1

A 60-year-old woman was transferred to the ER of a poison center in northern Iran from a local hospital. She and her sister (case 2) had ingested a plant in the forest that resembled wild lettuce. Following the consumption of the plant's raw leaves, the patient exhibited deliriant symptoms such as weakness, lethargy, and dizziness, which later escalated to restlessness, agitation, and confusion. The patient had a medical history of hypertension but no history of smoking, tobacco, or alcohol use. On initial examination, her pupils were dilated and reactive to light and her skin appeared warm and erythematous. Upon presentation to the ER, her vital signs were as follows: blood pressure 130/85 mmHg, temperature 37°C, heart rate 120 beats per minute, respiratory rate 20 breaths per minute, and oxygen saturation 97%.

### 2.2. Case 2

In addition to the first patient, a 71-year-old woman was also referred to the ER after consuming the same plant. Her symptoms included weakness, restlessness, and agitation. The patient had no significant medical history, and on initial examination, her pupils were dilated and her skin was warm. Her vital signs were as follows: blood pressure 110/70 mmHg, temperature 37°C, heart rate 90 beats per minute, respiratory rate 20 breaths per minute, and oxygen saturation 96%.

### 2.3. Diagnosis and Clinical Treatment

Upon admission of the two patients, standard protocols for the management of poisoned individuals were initiated, including oxygen therapy, intravenous line placement, and fluid resuscitation. Initial diagnostic assessments consisted of arterial blood gas analysis, electrocardiography, and routine laboratory testing.  Table 1 presents the laboratory results obtained upon admission to the ER for both cases.

**Figure 1:**
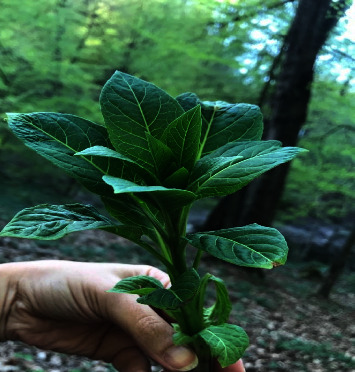
The plant, *Atropa belladonna*, that was eaten by the patients.

A photograph of the plant brought in by the patients' relatives was captured, along with several images taken by the family members at the location where the plant was found. The herbarium department of pharmacognosy at Mazandaran University of Medical Sciences in Sari, North Iran, identified the plant (as shown in Figure 1) as *Atropa belladonna*.

For the first patient, a single dose of haloperidol and midazolam was administered, and she was subsequently transferred to the intensive care unit. A midazolam drip was initiated at a dosage of 1 mg/hour, and after 12 hours, the patient's symptoms improved. Following 24 hours of treatment, during which the patient regained full consciousness, normal vital signs, and food tolerance, she was transferred to the poisoning ward. Ultimately, the patient was discharged from the hospital in stable condition.

For the second patient, midazolam was administered and her symptoms resolved, allowing for her discharge with full consciousness after 24 hours of observation. Both patients provided written informed consent for publication of this case report, and the study was conducted in accordance with the principles set forth in the Declaration of Helsinki. The study adhered to the CARE guidelines and methodology.

## 3. Discussion

We have presented a report of two patients who suffered from toxicity after inadvertently consuming *A. belladonna*. The reason for their consumption of the plant was confusion with the forest lettuce that is widely available near their residence in northern Iran. Although all parts of *A. belladonna* contain alkaloids, the leaves have the highest concentration. In both patients, the clinical symptoms observed were attributable to the ingestion of the leaves of the plant.

Our patients exhibited peripheral symptoms such as tachycardia and warm, red skin. The clinical manifestations of *A. belladonna* toxicity are primarily the result of the alkaloids' effects on the central and peripheral nerves, often leading to anticholinergic toxicity. The central effects of *A. belladonna* toxicity are dose-dependent and can include drowsiness, agitation, and short-term memory loss, picking motions with the hands, hallucinations, seizures, and coma. In contrast, the peripheral effects may include mydriasis with cycloplegia, dry mucous membranes, hyperreflexia, flushed skin, diminished bowel sounds or ileus, urinary retention, and hypertension or hypotension [8]. Our patients exhibited some of the important peripheral and central symptoms, including dilated pupils, weakness, restlessness, agitation, dizziness, and confusion.

One of our patients showed a significant increase in creatine phosphokinase (CPK) levels in laboratory findings. The mechanism underlying the increase in CPK levels caused by *A. belladonna* toxicity is not well defined. Some previous studies have suggested muscle breakdown and activation of intracellular calcium alkaline protease as potential causes for the increase in the CPK level [4]. Therefore, it appears that the increase in CPK levels is related to the severity of toxicity as well as agitation, which may require hospitalization in the intensive care unit (ICU).

*A. belladonna* can cause acute toxicity with varying manifestations, and its toxicity should be considered in the presence of an anticholinergic toxidrome. Depending on the severity of the symptoms, *A. belladonna* toxicity can be categorized as mild, moderate, or severe intoxication. Typically, the management of *A. belladonna* toxicity is conservative, and close hemodynamic monitoring in the ICU is recommended for patients with predominantly central symptoms [9].

Anticholinergic decontamination using activated charcoal may be effective if the ingestion occurred within the previous 2 hours, as activated charcoal can adsorb the toxic agents quite well. If the patient is highly agitated, benzodiazepines can be used for sedation. Hypotension should be managed with intravenous isotonic fluids, and in cases of persistent hypotension, vasopressor agents may be required. Due to the anticholinergic effects of *A. belladonna*, the use of drugs such as diphenhydramine and phenothiazines should be avoided.

Our patients exhibited neurological and deliriant symptoms such as lethargy, dizziness, agitation, and confusion. *A. belladonna* neurotoxicity has been associated with various psychotic symptoms, loss of consciousness, agitation, delirium, hallucinations, and seizures, as reported in the literature.  Table 2 provides some examples of clinical case reports and treatment methods for patients with *A. belladonna* neurotoxicity.

Physostigmine may be a useful treatment option to control both central and peripheral anticholinergic symptoms [16]. Physostigmine is a reversible cholinesterase inhibitor that can cross the blood–brain barrier [17]. Haloperidol is not considered effective for resolving central anticholinergic effects, but benzodiazepines have shown to be effective in treating agitation [18]. In our patients, conservative treatments were effective in reducing their symptoms, and therefore, physostigmine was not used.

Our cases involved the accidental consumption of *A. belladonna*, resulting in symptoms of both peripheral and central neurotoxicity. The patients were hospitalized and closely monitored in the ICU, where hydration and benzodiazepines were used to manage their symptoms. Further research on the neurotoxicity of *A. belladonna* is necessary to develop more effective treatment methods and to educate at-risk populations for future prevention [7].

## 4. Conclusion

*A. belladonna* toxicity can occur through accidental or intentional ingestion of any part of the plant, which contains toxic alkaloids. The resulting toxicity can lead to a range of severe anticholinergic conditions and symptoms. Supportive care is a crucial intervention in managing toxicity caused by this plant. For patients with neurological symptoms predominating, ICU monitoring would be appropriate. In cases of severe neurologic toxicity caused by anticholinergic symptoms associated with *A. belladonna* toxicity, treatment with physostigmine may be considered.

## Figures and Tables

**Table 1:** Initial laboratory data in the emergency room.

Parameter	Normal range	Result (case 1)	Result (case 2)
BS	90 to 110 mg/dL	130	110
BUN	6 to 20 mg/dl	13	10
Cr	0.5 to 1.3 mg/dL	1	0.8
K	3.5 to 5.5 mEq/L	4	3.8
Na	135 to 145 mEq/L	146	139
CPK	10 to 120 mcg/L	850	115
pH	7.35 to 7.45	7.37	7.42
HCO_3_	22 to 28 mmol/L	22.2	25.1
PCO_2_	35 to 45 mmHg	38.5	42.5

BS: blood sugar; BUN: blood urea nitrogen; Cr: creatinine; K: potassium; Na: sodium; CPK: creatine phosphokinase; pH: power of hydrogen.

**Table 2:** Some case reports, clinical characteristics, and treatment method of patients with *A. belladonna* neurotoxicity.

Study	Age/sex	Plant parts that have been eaten	Intention or purpose	Clinical signs and symptoms	Treatment method	Ref
Hasgül et al. (2020)	65/F	Fruits	Treatment of diabetes mellitus	Agitation, speech disorder, and consciousness change	Supportive treatment	(Hasgül et al., 2020) [10]
	58/M	Fruits	Treatment of diabetes mellitus	Impaired consciousness, agitation, difficulty in speaking, vomiting, and fever	Physostigmine 2 mg IV	
Cebeci et al. (2018)	8/F	Leaves	Accidentally eating the plant, next to spinach	Delirium, meaningless speech and visual hallucination	Physostigmine 0.02 mg/kg BD	(Cebeci et al., 2020) [11]
Glatstein et al. (2014)	20-day-old/M	Homeopathic medicine	Infantile colic	Generalized tonic–clonic seizures	Supportive treatment	(Glatstein et al., 2014) [12]
Demirhan et al. (2013)	49/F	Fruits	Accidentally	Delirium, agitation, and loss of consciousness	Supportive treatment	(Demirhan et al., 2013) [13]
Berdai et al. (2012)	11/F	Not reported	Treatment of jaundice caused by antituberculosis drugs	Confusion, incoherent speech, inability to recognize members of the family, and hallucinations	Symptomatic treatment and diazepam 5 mg BD	(Berdai et al., 2012) [14]
Fidan and Kırpınar (2011)	8/M	Fruits	Accidentally	Incoherent speech, attempting to catch invisible objects in the air, and inability to recognize family members	Supportive treatment and lorazepam 2.5 mg IV	(Fidan et al., 2011) [15]

## Data Availability

The data used to support the findings of this study are available from the corresponding author upon request.
